# Results from a randomized controlled trial of functional family therapy in Norway: effects on family functioning outcomes

**DOI:** 10.1186/s13034-026-01025-4

**Published:** 2026-01-17

**Authors:** Asgeir Røyrhus Olseth, Gunnar Bjørnebekk, Serap Keles, Kristine Amlund Hagen

**Affiliations:** 1 Norwegian Center for Child Behavioral Development, P.O. Box 7053, Majorstuen, Oslo, 0306 Norway; 2 Department of Special Needs Education, University of Oslo, Oslo, Norway; 3 Knowledge Centre for Education, University of Stavanger, Stavanger, Norway

**Keywords:** Functional family therapy, Youths, Family functioning, Disruptive behavior, Randomized controlled trial, Effectiveness trial

## Abstract

**Background:**

Several family factors are linked to later severe disruptive behavior problems, and family treatment is often recommended for treatment of adolescent disruptive behavior. Functional Family Therapy (FFT) is an evidence-based intervention designed to reduce adolescent disruptive behavior by improving family functioning. While prior research has evaluated the effectiveness of FFT in enhancing family functioning, there is limited evidence on its impact in service settings where treatment referral does not require prior involvement with the justice system.

**Methods:**

This parallel group, open label randomized controlled trial examined the short- and long-term effectiveness of FFT on family functioning outcomes. One hundred and sixty-one youths (*M*_*age*_ = 14.7, 45.9% female), referred to FFT in four Norwegian Child Welfare Service organizations for disruptive behavior, were randomly assigned to FFT (*n* = 88) or treatment as usual (TAU; *n* = 73). The outcomes were parent- and youth-reported cohesion and conflict in the family; parent- and youth-reported positive involvement and positive parenting, parent-reported conflict resolution strategies (negotiation and psychological aggression); and youth-reported social support from parents and attachment (trust, communication, and alienation). Data were collected at fixed timepoints before treatment (pretest), six months after pretest (posttest), and 18 months after pretest (follow-up). A latent change score modeling approach was employed to assess differential change following treatment.

**Results:**

Results showed no intervention effect of FFT compared to TAU on any outcome. Improvements were found between pretest and posttest for the entire sample, regardless of treatment group, on parent-reported cohesion (*d* = −0.27) and conflict (*d* = 0.30); and youth-reported social support (*d* = −0.33), cohesion (*d* = −0.23), conflict (*d* = 0.25*)*, and alienation (*d* = 0.33). Between posttest and follow-up significant improvements were found on youth-reported cohesion (*d* = 0.29) and conflict (*d* = −0.32).

**Conclusion:**

Findings did not support the hypothesized superiority of FFT over TAU in the Norwegian context.

ISRCTN trial registry number and date of registration (retrospectively registered): ISRCTN58861782, May 24th 2013.

**Supplementary Information:**

The online version contains supplementary material available at 10.1186/s13034-026-01025-4.

## Background

Decades of research underscore the importance of family factors in the development and maintenance of adolescent disruptive behavior (e.g., [19, 28]). The strength and quality of the parent-youth relationship can act either as a risk or protective factor for adolescent delinquency. For example, poor parental attachment is associated with increased youth delinquency [17], whereas youths who experience supportive parenting, characterized by acceptance, warmth, trust, open communication, and the absence of hostility and rejection, are less likely to engage in delinquent behavior [16]. Family systems theory emphasizes the importance of viewing and treating the family as a whole rather than mere individuals or limited to bi-directional relationships [9]. From an interactional perspective, [39] describes the development of youth delinquency as a “reciprocal and dynamic” (p. 2594) process whereby family attachment interacts with youth delinquent behavior over time either prolonging or curbing delinquency. For example, weak bonds to family or school may allow for the development of delinquency, but youths’ involvement in delinquency is also likely to further weaken these bonds over time [39].

The extensive literature linking family functioning to the development and maintenance of severe problem behavior has led to the development of several family therapeutic interventions, and family therapy is widely recommended for treating adolescent disruptive behavior (see for example [5, 22]). Functional Family Therapy (FFT) is one such family treatment, developed through research and therapeutic practice over the last 50 years, that specifically targets dysfunctional family functioning to help reduce youth disruptive behavior [2, 33]. The family is the primary focus of the intervention, and the goal in treatment is to assist families in altering their functioning through reducing intrafamilial risk factors and to promote protective factors, thereby also reducing the effects of potential risk factors in the youth’s and family’s environment [1]. The long-term goal of FFT is to enhance parents’ ability to successfully support their adolescents through the transition to young adulthood, and to create a family environment where the youths perceive their family as relevant and supportive during this transition [2]. The treatment is delivered in five phases: engagement, motivation, relational assessment, behavior change, and generalization. These phases contain intervention elements that are expected to have a cascading effect, ultimately contributing to a collective treatment effect. Several aspects of family functioning are targeted in the different phases: Family negativity and conflict are addressed with specific therapeutic practices in the engagement phase (e.g., change-focus and change-meaning techniques; [2] pp. 92–111) as these family factors are expected to be barriers for both retaining families in treatment and later treatment success. In later stages, the intervention also focuses on improving family interactions through training relevant skills (e.g. effective communication, problem solving, and negotiation; [2] pp. 137–150).

With respect to the evidence base for FFT’s effectiveness and its dissemination (i.e., implementation in different real-world service contexts), research findings presented in recent years present a mixed picture. The developers of FFT point to dissemination research supporting that FFT can be implemented in community settings that vary with respect to reasons for referral (e.g., delinquency) or sources of referral (e.g., child welfare and mental health; [33] pp. 547–548). [22], however, in a recent evidence-base update on interventions for adoescent disruptive behaior problems, classified FFT as belonging to the group of interventions with the highest level of evidence (i.e., well-established) for juvenile justice involved youths. For youths without juvenile justice involvement, no interventions were identified at the highest level of evidence, and FFT was classified as belonging to interventions described as possibly efficacious (third level; [22] p. 14).

In addition to this evidence-base update, in a recent systematic review and meta-analysis [21] synthesized findings across a broad set of outcomes, including family functioning. Drawing on 15 studies, they reported substantial variability both across and within studies on different outcomes and concluded that “FFT is not consistently more (or less) effective than the active treatments to which it has been compared” (p. 43). In their review, [21] identified five studies examining effects of FFT on family functioning outcomes, comprising four randomized controlled trials and one quasi-experimental study. The analysis revealed no significant differences between FFT and control conditions across 60 outcome measures. The studies included in the analysis varied with respect to target groups and control conditions. Three studies focused on adolescents in the juvenile justice system with various active control treatments: [18] compared FFT to supervised probation, [13] to probation and a non-evidence-based family intervention, and [6] to individual therapy or mentoring through probation services. Two additional studies examined FFT in non-juvenile justice settings: [32] studied hyperactive youths, comparing FFT to group therapy and a waitlist control, while [36] investigated FFT for runaway, alcohol-abusing youths, comparing it to ecologically based family therapy and services as usual. These studies also differed in how they measured family functioning. Three included both parent and youth reports, [6] relied on therapists’ reports, and [36] used only youth reports. Sample sizes varied, ranging from 18 [32] to 129 [13]. Four of the five studies found no significant difference in family functioning outcomes, but [32] reported FFT’s superiority over both control groups in family effectiveness and over the waitlist control group on emotional distance. Additionally, three studies [6, 18, 36] found significant improvements in family functioning over time. It is also worth noting some issues with respect to the relevance and quality of the evidence regarding FFTs’ effects on family functioning. The sample sizes in these five studies were generally small, and the only study showing between-group effects was conducted early in the 1980 s, possibly making generalization to treatment and the target group today tenuous. [21] scored the risk of bias in these five studies, and all of them had high risk on two or more criteria (p. 22). They also concluded that the certainty of the available evidence is very low across all studies included in the meta-analysis [21].

The systematic review conducted by [21] and the evidence-base update conducted by [22] suggests two important issues with the literature on FFT-treatment: few studies have examined FFT’s effectiveness in changing family functioning outcomes, a core assumption in the treatment model’s theory of change, and, the majority of studies that has done so was conducted in contexts where juvenile justice involvement was a requirement for receiving treatment. This suggests that there is a need for research evidence examining FFT’s effectiveness with respect to altering family functioning, and whether or not such effects generalize across different contexts.

## The current study

The aim of the current study was to examine effectiveness of FFT in the Norwegian Child Welfare Services (CWS), a context where the treatment was voluntary, and involvement with the justice system was not a requirement for receiving FFT, and to contribute to the knowledge base on FFTs effectiveness under such conditions. The family functioning outcomes covered in this article were specified as secondary outcomes in the trial registration, and supplement results on primary outcomes—parent- and teacher-reported aggressive behavior, rule-breaking behavior, internalizing problems and social skills; youth self-reported delinquency and association with negative peers; and teacher-reported academic performance and adaptation at school—reported elsewhere (see [31]). When examining primary outcomes, no significant short-term or long-term effects were found in favor of FFT over treatment-as-usual control group (TAU), and one significant long-term effect was found on internalizing behavior favoring TAU.

In this article, we address the research question “How effective is Functional Family Therapy, compared to general family counselling, in reducing negative communication and conflicts and increasing positive communication, social support, cohesion, and the quality of relations and conflict tactics in the family?” [30] study hypothesis section). Based on FFT’s specific treatment elements directed at, and strong focus on, altering family functioning as an intermediate treatment goal, we hypothesized that FFT would produce better effects than TAU on these outcomes.

To examine this hypothesis, we conducted intention to treat (ITT) analyses of 159 families randomly assigned to either FFT or TAU to examine between-group effects of the treatment. We also modeled the average change over time for the total sample, regardless of the treatment, as this may inform us about potential changes related to treatment in the overall group in the CWS.

## Method

Details regarding this study have been extensively described in a previous publication (see [31]) and were briefly described here.

### Participants and recruitment

Participants were 161 youths and their families. Mean youth age was 14.7 years (*SD* = 1.47), and there were 45.9% females^1^. Parents’ mean age was 44 years (*SD* = 6.9), and most of them (*n* = 103, 67.3%) were families with parents divorced or living apart. Forty youths (26.1%) were living with parents that were living together, while ten youths (6.5%) were living in long-term foster care or with adoptive families, and they received treatment with their foster- or adoptive families. Six youths’ information on family situation was missing.

Participants were recruited from families referred to four CWS sites for severe adolescent disruptive behavior during the period between 2013 and 2017. The services that took part were located in the southern, eastern, and central parts of Norway. Assessment for inclusion or exclusion into the study was conducted by the leaders of the FFT teams based on the information received as part of the referral process. Inclusion criteria^2^ for the study were youths aged 11 to 18 who had committed a criminal offence and were at risk of recidivism; or exhibited severe disruptive behavior in the home, school, or community (e.g., aggressive or violent behavior; vandalism; drug abuse^3^, or other severe rule violations); or were at risk of out-of-home placement due to behavioral problems. Youths showing problems such as verbal aggression (e.g., threats) or truancy could also be included if these were related to the above-mentioned problem behaviors. Exclusion criteria were if the youths had no primary caregivers (e.g., living alone or in institutional care); youths with autism, psychosis or showing acute suicide risk; families where the local CWS were conducting inquiries into potential ongoing abuse or neglect; homes that could pose risk to therapist health and safety; and cases where there were ongoing treatment that could interfere with FFT (see also [30] Eligibility section). Due to economic constraints in the research project, families who lived more than one hour drive from the CWS offices, or needed interpreters to answer questionnaires, were excluded from the study. The two most frequent primary reasons for referrals of those included were verbal conflicts at home (*n* = 57, 35.8%) and violent behavior at home (*n* = 32, 20.1%).

The study was planned with a sample size of *N* = 250 based on a medium effect size (*d* = 0.5), an $\:\alpha\:$-level = 0.05, β = 0.95 and 20% oversampling to meet potential drop-out. Recruitment was terminated before reaching the planned sample size due to the issues with therapists’ turnover, two treatment sites being discontinued, and problems recruiting families to the study [31].

### Alterations made to recruitment and enrollment procedures

Due to the recruitment problems, changes were made to the inclusion criteria intake procedures and to TAU approximately one year into the study. These alterations were reported in detail in [31], but one important alteration should be reiterated here as it has a direct bearing on interpretation of results. This study was initially designed with TAU provided exclusively by the Family Counselling Service (FCS). The FCS is a government-funded organization offering support to families in crises (e.g., divorce, family conflict; see also Norwegian Directorate for Children, Youth and Family Affairs [29]. During the first year of the study, recruitment at several of the participating sites was slower than expected. When examining enrollment reports from FFT-leaders, we saw that, in addition to families not consenting to participate, a substantial number of families that met FFT inclusion criteria were excluded due to being at high risk or in a crisis situation. A further examination of this indicated problems with the FCS’s capacity to take on these families. This prompted a protocol change to also permit TAU to be delivered through CWS. This modification in turn introduced Multisystemic Therapy (MST), which is a high intensity evidence-based intervention, as an available treatment within the control condition, and the intake procedures were revised to ensure that families identified as being in crisis and randomly assigned to the control group could receive MST (i.e., MST capacity to take on cases was checked before random assignment).

### Design and research procedures

This study was a two-arm RCT (1:1 allocation ratio). Random assignment was conducted by staff at Norwegian Center for Child Behavioral Development (NCCBD) after inclusion and pretest and was based on separate randomization sequences for each of the sites. Randomization sequence was generated by the data manager at NCCBD using www.random.org [14] and was concealed from staff in the CWS and FFT-leaders responsible for assessing eligibility and inclusion or exclusion, but not from staff at NCCBD tasked with random assignment. Results of the random assignment were communicated from NCCBD to the FFT-leaders who in turn referred families either to FFT-treatment or to TAU.

**Fig. 1:**
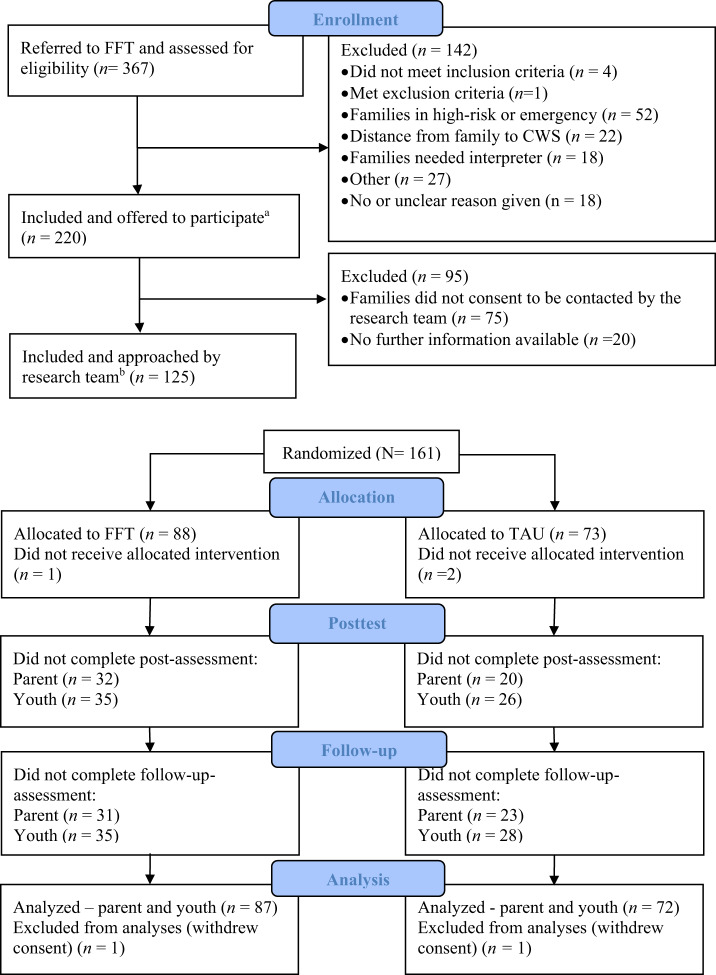
CONSORT participant flowchart. CONSORT = Consolidated Standards of Reporting Trials. ^a^Five cases were registered as referred, but no decision about inclusion or exclusion was recorded. ^b^For one of the sites, reporting of enrollment for the period 2015-2017 was incomplete. Therefore, the number of randomized families was higher than the number of families registered as included in the study

Data collections were conducted at fixed intervals at three different time points: immediately before treatment started (pretest), six months after the treatment started (posttest), and 18 months after the treatment started (12-month follow-up). Parent and youth responses were collected using self-administered computerized or pen and paper questionnaires. Data collections were conducted in the homes of the families or at the offices of the CWS, and trained staff from NCCBD were present to inform the family about the study, collect consent forms, and render technical support if needed. Families were given a gift card worth approximately USD 38 per data collection. Blinding or masking treatment conditions were not possible. One primary parent respondent was selected per family in families where more than one parent participated. The parent that provided responses at the most data collection time points was selected. If both parents had an equal number of responses, mother’s data was chosen. Figure 1 shows CONSORT flow chart [35] with information regarding enrollment, inclusion, and retention rates throughout the study. The study design and hypotheses were registered in ISRCTN (ISRCTN58861782) approximately one month into recruitment (May 24th 2013): 10.1186/ISRCTN58861782.

### Treatment conditions

#### Functional family therapy

FFT treatment was offered by therapists employed at CWS and consisted primarily of 60 min sessions with youths and parents together at the CWS offices or in the family’s homes. Treatment lasted on average 12.7 (*SD* = 6.8) hours over a period of 20.9 weeks (*SD* = 8.7). Between sessions, homework and tasks were assigned. FFT was delivered by trained FFT-therapists, and these were organized in specific teams within the different CWS. FFT therapists received specific training offered by Norwegian implementation effort was led by NCCBD with support from the US based organization FFT limited liability company (LLC). Clinical fidelity and dissemination adherence were assessed by FFT supervisors as part of the regular implementation of FFT. These supervisors were experts in the FFT treatment and assessed therapist adherence to core concepts and how competently treatment was delivered (clinical fidelity), and to what degree a set of tasks and procedures specific to FFT was present in treatment (dissemination adherence). Both were scored on a 0 to 6 scale, and the average scores were calculated per site per quarter [34]. The overall average clinical fidelity score for participating services was 4.4 (*SD* = 0.2), and dissemination adherence score was 4.7 (*SD* = 0.2). These scores are above criteria set by FFT LLC (M.S. Robbins, personal communication, March 31, 2023), as indications of acceptable implementation adherence and clinical fidelity.

#### Control condition

Treatment for the control group, TAU, was offered by other therapists that were working in the participating CWSs or the FCSs. No restrictions were put on the type of treatment families randomized to control group could receive other than that they could not receive FFT. The most frequent types of control treatments were Multisystemic Therapy (MST; 40.3%), parent training (26.4%) and family counselling (8.3%). Information about the treatment was missing for 10 families (13.9%). Two families (2.8%) received no treatment, and two (2.8%) were referred to treatment in the national child and youth psychiatric services. MST was available in three of the participating sites, and the FFT inclusion criteria partly overlapped with the criteria for receiving MST. Three families in the control group were recorded as having received FFT treatment contrary to their allocation. One case received FFT between the pretest and the posttest, while two cases received FFT between the posttest and the follow-up.

### Assessments and instruments

#### Alabama parenting questionnaire

Alabama Parenting Questionnaire (APQ) is an instrument assessing five dimensions of parenting that are expected to be related to the development and treatment of externalizing problems in children [11]. The two subscales used in the current study were: positive parenting (6 items) and involvement (10 items) reported by both youths and parents. Items were scored on a five-point scale (1 = *never* to 5 = *always*). Higher scores indicated higher levels of positive parenting and involvement. The internal consistency on the different time points ranged from α = 0.89 − 0.92 for positive parenting and α = 0.86 − 0.90 for involvement on youth report, and α = 0.76 − 0.77 for positive parenting and α = 0.72 − 0.79 for involvement on parent report.

#### Family environment scale

Family Environment Scale (FES) is a comprehensive instrument covering 10 different aspects of family’s social environment [26]. In the current study, the subscales for cohesion (9 items) and conflict (9 items) in the family were used. These were reported by both parents and youths. Items were scored dichotomously (0 = *false* or 1 = *true*). Higher scores indicated higher levels of cohesion and conflict. The internal consistency of the subscales ranged from α = 0.72 − 0.82 and α = 0.73 − 0.77 for youth-reported cohesion and conflict, and from α = 0.64 − 0.73 and α = 0.72 − 0.76 for parent-reported cohesion and conflict, respectively.

### Inventory of parent and peer attachment

Inventory of Parent and Peer Attachment (IPPA) is a youth self-report measure assessing attachment between youths and their parents and peers [3]. In the current study, we used the subscales for trust (10 items), communication (9 items) and alienation (6 items) between youth and the selected primary parent respondent. Items were scored on a five-point scale (1 = *almost never or never true* to 5 = *almost always or always true*). Higher scores on the three subscales indicated higher degree of trust, communication, and alienation. The internal consistency of the subscales ranged from α = 0.89 − 0.91, α = 0.85 − 0.87 and α = 0.77 − 0.84 for trust, communication, and alienation, respectively.

### The revised conflict tactics scale

The Revised Conflict Tactics Scale (CTS2) measures several aspects of how conflict is dealt with in families [37]. Parent-report on the subscales for psychological aggression (8 items) and negotiation (6 items) was used in the current study. Psychological aggression covers non-physical aggressive acts (both verbal and non-verbal), and negotiation describes to what degree disagreement is settled through discussion [37] p. 289). Reference time frame was set at four months to primarily examine changes occurring between pretest and posttest. Items were scored on a frequency scale based on the chronicity scoring suggested by [37] 0 = *never/not in the past 4 months*,* but happened earlier* to 25 = *more than 20 times in the past 4 months*). Higher scores reflected a higher number of occurrences of psychological aggression and negotiation in the past four months. The internal consistency on the different time points ranged from α = 0.73 − 0.81 for negotiation, and α = 0.56 − 0.75 for psychological aggression.

### Social support

Youths perceived social support in their family was measured using a scale developed by Ulriksen et al. [40] based on Cohen and Willis’ [8] theoretical framework. The scale consisted of five items containing statements about attachment between youth and parents, available social support, and mutual care in the family. The items were scored on a four-point Likert-type scale (1 = *completely disagree* to 4 = *completely agree)*. Higher scores reflected a higher degree of perceived social support. Internal consistencies at the different timepoints were in the range of α = 0.89 − 0.94.

### Statistical analyses

Change over time, and the difference in change between TAU and FFT, were examined using latent change scores within a latent curve modeling (LCM) framework as described by [27]. This approach allows for modeling average change and between-group differences in change while appropriately handling intraindividual differences in change. It also offers the option of anchoring at posttest and thus allowing the change in two different time periods (i.e., pretest to posttest, and posttest to follow-up) to be estimated separately in the same model [27] p. 320).

Modeling was conducted in Mplus separately for each outcome, and in two steps: First, we modeled change in the combined total sample (unconditional models). Second, we included treatment as a predictor on latent change scores and the intercept (conditional models). When examining treatment effects, we focused primarily on the parameter estimate of the treatment condition (TAU = 0 and FFT = 1) on differences in change scores. Effect sizes (ES; Cohen’s *d*; [7] were calculated for pretest to posttest change, and posttest to follow-up change in the combined sample, and for between-group difference in these change scores (see supplemental information SI1 for details on ES calculation). All cases were analyzed as belonging to the groups they were randomly assigned in line with ITT-principles [23]. Missing data were handled using multiple imputation (MI) with auxiliary variables in Mplus using the sequential regression model option [4]. A list of software used in analyses is available in SI2.

## Results

### Results of the preliminary data analyses

Table 1 contains samples sizes, means and standard deviations for all outcome variables at pretest, posttest, and follow-up for the two different groups. No significant pretest differences were found on any of the outcome variables. Rates of missing on outcome variables in the models ranged from 2.5% to 64.8%. The responses to the conflict tactics scale exhibited particularly high missing rates, ranging from 40.3% to 64.8% at each time point. Psychological aggression was in addition severely non-normal, and the attempt to model this outcome with the LCM approach was not feasible (see SI3 for more information). When examining missing over time, 97 (61%) of the sample had parent-reported outcomes on all timepoints, and 84 (52.8%) had youth-reported outcomes on all timepoints. Due to the amount of missing data and attrition, we conducted missing analyses focusing on specific patterns of missingness over time classified as either monotone or ignorable missingness (see SI4 for tables showing all missing patterns and classified missing patterns). Categorical variables (youth gender, immigrant background and treatment site) were examined in χ^2^ tests. For continuous outcomes, we examined effect sizes between those present or missing at either posttest or follow-up. Potential systematic attrition related to intervention was examined in two-way ANOVA analyses (treatment group*missing group).

**Table 1:** Descriptive statistics for outcomes per treatment group and by time point

Outcome	Pretest				Posttest				Follow-up			
	FFT		TAU		FFT		TAU		FFT		TAU	
	*M (SD)*	*n*	*M (SD)*	*n*	*M (SD)*	*n*	*M (SD)*	*n*	*M (SD)*	*n*	*M (SD)*	*n*
*Parent-reported outcomes*												
Positive involvement	36.29 (5.07)	80	36.16 (4.51)	69	36.57 (5.54)	54	37.15 (5.03)	53	36.36 (5.59)	55	36.23 (4.77)	48
Positive parenting	24.3 (3.38)	84	24.19 (3.22)	70	24.29 (3.52)	55	24.09 (3.32)	53	24.26 (3.17)	57	23.90 (3.39)	50
Negotiation	24.26 (29.27)	43	26.10 (28.60)	50	25.47 (25.74)	30	24.11 (23.50)	35	30.50 (28.33)	28	23.86 (24.66)	28
Psychological aggression	3.14 (5.38)	44	4.02 (8.94)	51	2.80 (5.98)	30	3.29 (13.95)	35	4.10 (9.81)	30	2.75 (7.57)	28
Cohesion	6.07 (2.40)	83	5.90 (2.25)	69	6.32 (2.26)	53	6.77 (2.03)	53	6.93 (1.94)	55	6.81 (1.79)	48
Conflict	4.01 (2.40)	83	3.65 (2.41)	69	3.34 (2.46)	53	2.71 (1.94)	52	2.84 (2.19)	55	2.77 (1.99)	48
*Youth-reported outcomes*												
Positive involvement	28.28 (7.79)	82	30.12 (9.56)	68	28.33 (9.39)	52	30.22 (10.47)	46	29.75 (8.88)	48	29.89 (11.33)	44
Positive parenting	18.96 (6.25)	83	18.70 (6.58)	69	18.31 (6.49)	52	20.42 (7.24)	45	19.94 (6.82)	50	19.86 (6.98)	44
Social support	2.83 (0.81)	85	2.75 (0.82)	70	3.00 (0.85)	52	3.11 (0.82)	47	3.07 (0.88)	49	3.17 (0.87)	44
Cohesion	4.76 (2.54)	83	5.06 (2.39)	69	5.56 (2.27)	52	5.48 (2.49)	46	6.54 (2.35)	48	5.86 (2.88)	43
Conflict	4.43 (2.57)	84	4.06 (2.34)	68	4.13 (2.50)	52	3.17 (2.68)	46	2.84 (2.44)	49	2.73 (2.25)	44
Alienation	15.22 (5.26)	83	15.11 (5.82)	70	13.73 (4.98)	52	12.79 (5.75)	47	13.42 (5.90)	52	12.76 (5.82)	45
Communication	27.20 (8.03)	82	27.99 (9.59)	70	27.83 (8.92)	52	29.32 (8.68)	47	29.73 (8.67)	52	30.16 (9.46)	45
Trust	34.01 (8.98)	81	34.86 (10.62)	69	34.65 (10.30)	52	37.45 (9.75)	47	38.38 (7.59)	52	36.62 (10.78)	45

Results of the missing analyses showed that females had a higher risk of being missing in the total sample χ^2^ (2, *N* = 159) = 6.81, *p* =.03, and several pretest differences were found based on a criterion of *d* > 0.2 suggesting that these could potentially be related to missingness [10]. Furthermore, we found a significant interaction between intervention group and missingness for the youth-reported parental involvement, *F*(2, 159) = 4.62, *p* =.01, suggesting potential differential attrition in the two treatment groups. As a result of the missing analyses, sets of auxiliary variables were selected and included in the MI to make a conditional missing-at-random assumption [10] more plausible (see SI5 for more details). Two hundred imputed datasets were created per outcome. Imputations were conducted separately for each treatment group to allow for potential differences in missing mechanism [38].

### Estimates of overall change—unconditional LCMs

Model fit indices and any model modifications are presented in SI6. Parameter estimates from unconditional models examining average change in the total combined sample are available in Table 2. From pretest to posttest (change 1), results showed that two of the six parent-rated outcomes, and four of the eight youth-rated outcomes changed significantly^4^. Parent-reported cohesion and conflict changed significantly in a positive direction (*d* = −0.27 and *d* = 0.30, respectively). Of the youth-reported outcomes, significant positive changes were found on social support (*d* = −0.33), cohesion (*d* = −0.23), conflict (*d* = 0.25*)*, and alienation (*d* = 0.33).

**Table 2:** Unconditional LCMs – latent variable means and standardized effect sizes of change parameters

Outcomes	Level at posttest (intercept)	Change between pretest and posttest (Change 1)		Change between posttest and follow-up (Change 2)	
	Latent mean (SE)	Latent mean (SE)	Cohen´s *d* [95% CI]	Latent mean (SE)	Cohen´s *d* [95% CI]
*Parent-reported outcomes*					
Positive involvement^a^	36.69*** (0.49)	− 0.48 (0.47)	− 0.07 [− 0.19, 0.06]	− 0.37 (0.55)	− 0.05 [− 0.20, 0.10]
Positive parenting	24.03*** (0.30)	0.21 (0.29)	0.07 [− 0.11, 0.24]	− 0.09 (0.32)	− 0.03 [− 0.22, 0.16]
Negotiation	24.93*** (2.87)	− 0.51 (3.36)	− 0.02 [− 0.24, 0.21]	1.83 (4.09)	0.06 [− 0.21, 0.34]
Cohesion	6.66*** (0.20)	− 0.67** (0.22)	− 0.27 [− 0.45, − 0.09]	0.23 (0.19)	0.09 [− 0.06, 0.24]
Conflict	3.10*** (0.20)	0.75*** (0.20)	0.30 [0.14, 0.46]	− 0.30 (0.18)	− 0.12 [− 0.26, 0.02]
*Youth-reported outcomes*					
Positive involvement	29.51*** (0.99)	− 0.40 (1.02)	− 0.05 [− 0.27, 0.18]	0.52 (1.19)	0.06 [− 0.20, 0.32]
Positive parenting	19.11*** (0.68)	− 0.18 (0.70)	− 0.03 [− 0.23, 0.18]	0.47 (0.77)	0.07 [− 0.16, 0.30]
Social support	3.06*** (0.08)	− 0.26** (0.08)	− 0.33 [− 0.54, − 0.12]	0.02 (0.12)	0.03 [− 0.27, 0.32]
Cohesion	5.49*** (0.24)	− 0.58* (0.27)	− 0.23 [− 0.45, − 0.02]	0.71* (0.32)	0.29 [0.04, 0.53]
Conflict	3.64*** (0.26)	0.61* (0.27)	0.25 [0.03, 0.47]	− 0.79** (0.30)	− 0.32 [− 0.56, − 0.08]
Alienation	13.35*** (0.54)	1.84** (0.58)	0.33 [0.12, 0.54]	− 0.04 (0.67)	− 0.01 [− 0.25, 0.23]
Communication	28.67*** (0.85)	− 1.10 (0.86)	− 0.12 [− 0.32, 0.07]	1.35 (1.00)	0.15 [− 0.07, 0.38]
Trust	35.86*** (1.01)	− 1.28 (1.10)	− 0.13 [− 0.35, 0.09]	1.57 (1.17)	0.16 [− 0.08, 0.40]

Unstandardized parameter estimates. *N* = 159. CI = confidence interval. Posttest specified as intercept. Change 1 parameter estimates were calculated as pretest – posttest. Change 2 parameter estimates were calculated as follow-up - posttest. A negative latent means/ES for change 1 indicates an increase, and a positive one indicates a decrease between pretest and posttest. For Change 2, a negative latent means/ES indicates a downward change, and a positive parameter estimate indicates an upward change. See SI1 for a description of how Cohen’s *d* was calculated

^a^For this model, correlations between time specific residual variances at pretest and follow-up were freely estimated

*p <.05. **p <.01. ***p <.001

Between posttest and follow-up (change 2), significant changes were found on youth-reported cohesion (*d* = 0.29) and conflict (*d* = −0.32). All significant changes were in the direction indicating better functioning after treatment for the whole sample.

### Estimates of treatment effect—conditional LCMs

Parameter estimates for the difference in the change between FFT treatment and TAU are presented in Table 3. No significant differences were found between the two groups in any of the change scores^5^.

**Table 3:** Conditional LCMs – regression coefficients and standardized effect sizes of treatment on level at posttest and change scores

Outcome	Level at posttest (Intercept)		Change between pretest and posttest (Change 1)		Change between posttest and follow-up (Change 2)	
	*B (SE)*	Cohen´s *d* [95% CI]	*B (SE)*	Cohen´s *d* [95% CI]	*B (SE)*	Cohen´s *d* [95% CI]
*Parent-reported outcomes*						
Positive involvement^a^	− 1.22 (0.96)	− 0.17 [− 0.43, 0.09]	1.28 (0.90)	0.18 [− 0.07, 0.43]	1.22 (1.05)	0.17 [− 0.12, 0.46]
Positive parenting	− 0.25 (0.61)	− 0.08 [− 0.44, 0.29]	0.35 (0.59)	0.11 [− 0.24, 0.46]	0.58 (0.69)	0.18 [− 0.23, 0.59]
Negotiation	1.14 (5.82)	0.04 [− 0.35, 0.43]	− 2.85 (6.83)	− 0.10 [− 0.56, 0.36]	0.34 (8.14)	0.01 [− 0.54, 0.57]
Cohesion	− 0.59 (0.39)	− 0.24 [− 0.55, 0.07]	0.74 (0.44)	0.30 [− 0.05, 0.65]	0.54 (0.38)	0.22 [− 0.09, 0.53]
Conflict	0.86* (0.39)	0.35 [0.04, 0.66]	− 0.51 (0.39)	− 0.20 [− 0.51, 0.10]	− 0.58 (0.36)	− 0.24 [− 0.52, 0.05]
*Youth-reported outcomes*						
Positive involvement	− 2.51 (1.97)	− 0.28 [− 0.72, 0.15]	0.69 (2.03)	0.08 [− 0.37, 0.52]	1.16 (2.42)	0.13 [− 0.40, 0.66]
Positive parenting	− 2.40 (1.35)	− 0.36 [− 0.77, 0.04]	2.68 (1.40)	0.41 [− 0.01, 0.83]	1.50 (1.59)	0.23 [− 0.25, 0.70]
Social support	− 0.11 (0.16)	− 0.14 [− 0.54, 0.27]	0.18 (0.17)	0.24 [− 0.19, 0.66]	− 0.00 (0.23)	− 0.01 [− 0.57, 0.56]
Cohesion	− 0.08 (0.48)	− 0.03 [− 0.41, 0.35]	− 0.16 (0.54)	− 0.07 [− 0.49, 0.36]	0.68 (0.63)	0.27 [− 0.22, 0.77]
Conflict	0.95 (0.51)	0.39 [− 0.02, 0.80]	− 0.61 (0.54)	− 0.25 [− 0.69, 0.18]	− 0.69 (0.61)	− 0.28 [− 0.77, 0.21]
Alienation	1.01 (1.12)	0.18 [− 0.22, 0.58]	− 0.81 (1.20)	− 0.15 [− 0.58, 0.28]	0.05 (1.43)	0.01 [− 0.50, 0.52]
Communication	− 2.28 (1.75)	− 0.26 [− 0.65, 0.13]	1.42 (1.77)	0.16 [− 0.23, 0.55]	1.15 (1.88)	0.13 [− 0.29, 0.55]
Trust	− 2.96 (2.04)	− 0.30 [− 0.72, 0.11]	2.15 (2.23)	0.22 [− 0.23, 0.67]	3.93 (2.30)	0.40 [− 0.06, 0.87]

Unstandardized parameter estimates. *N* = 159. CI = confidence interval. Treatment variable coded TAU = 0, FFT = 1. See SI1 for a description of how Cohen’s *d* was calculated

^a^For this model, correlations between time specific residual variances at pretest and follow-up were freely estimated

*p <.05. **p <.01. ***p <.001

## Discussion

The main goal of this study was to examine the effects of FFT on family functioning in a Norwegian CWS context. Contrary to our expectations, the findings did not provide support for the hypothesized superiority of FFT over TAU. We also examined the change in the total sample consisting of both FFT and the control group, and found significant average changes indicating better functioning after treatment on six out of 14 outcomes from pretest to posttest, and two out of 14 posttest to follow-up: Both parents and youths reported positive changes in cohesion and conflict between pretest and posttest, and youths reported on average further improvement in the 12-month period after posttest (follow-up) on these two outcomes. Youths also reported improvements between pretest and posttest on alienation and social support. There were no significant negative changes during the follow-up period, suggesting that positive changes were sustained over time independent of the type of treatment. The magnitude of the changes varied between *d*=−0.33 and *d* = 0.33, and these are considered small effect sizes by conventional standards [7]. The results on family functioning outcomes were also similar to those we found on primary outcomes in this study: We found no significant differences on parent- and teacher-reported disruptive behavior and school performance, and one delayed effect was found in favor of the control group on internalizing problems [31].

Our findings align with recent literature regarding FFT’s effectiveness on family functioning as reported by [21]. Specifically, FFT did not outperform an active control group, similar to results from earlier studies comparing FFT to regular supervised probation [18], ecological family therapy [36], and non-evidence-based family therapy [13]. Our findings did however contrast those reported by [32] where significant effects of FFT were found on family effectiveness compared to both a control group receiving group therapy and a waitlist control group, and on the emotional distance between youths and their parents compared to the waitlist group.

The absence of evidence supporting the hypothesized superiority of FFT over the control group in this study suggests that FFT is not more effective than TAU within the Norwegian context. One possible explanation, related to both the service context and the target population, is that the Norwegian target population may differ on family functioning from those settings where youths are referred to treatment only after committing criminal offenses. In such contexts, the proportion of youths displaying early-onset behavior problems, consistent with [24] classification of life-course persistent (LCP) antisocial behavior, is likely to be higher. Findings from the Dunedin Study also indicate that youth in the LCP group have higher levels of family risk compared to those whose antisocial behavior begins in adolescence [25]. Consequently, the lack of significant differences in the current study, despite FFT’s strong emphasis on improving family functioning, may point to the possibility that FFT is better suited to families with more severe impairments in family functioning, and that the present sample included fewer such families.

However, several factors should be considered when interpreting this finding. Most notably, approximately 40% of participants in the control group received Multisystemic Therapy (MST), a high-intensity, evidence-based intervention targeting many of the same risk factors as FFT (see [15]). A meta-analysis by [20] demonstrated MST’s effectiveness in improving family functioning outcomes. Because the current study was not designed to compare FFT directly with MST, the superiority hypothesis might not have been formulated if it had been known that such a substantial proportion of the control group would receive MST. A second possibility, also noted elsewhere [31], concerns the severity of problems in the target population for this study. Because our sample was drawn from families eligible for FFT in regular services, primary referral reasons included relatively low-level difficulties such as verbal aggression and truancy. Although we lack normative data to compare family functioning scores directly with the general population, previous analyses indicate that the sample displayed lower-than-expected levels of aggressive and rule-breaking behavior relative to FFT’s intended target group [31]. Consequently, it is possible that some families entered treatment with only minor impairments in family functioning, leaving limited room for detecting differences in improvement between the two conditions.

The change in the combined sample (i.e., unconditional models) also warrants further discussion. There were significant improvements in the overall sample regardless of which treatment they received. As they are within-group change estimates, the resulting effect sizes cannot be directly compared with effect sizes derived from between-group analyses, but they may still offer an insight into change for youths and families who have received intensive treatment in the Norwegian CWS. With respect to both cohesion and conflict, families were functioning better after treatment, and this was reported by both parents and youths. In addition, youths reported a further improvement in these aspects of their families functioning in the long-term, during the 12-month follow-up period. A reduction in conflict reported both by youths and their parents may be important as mutual parent-child hostility may be linked to externalizing problems [12]. A reduction in youths feeling of alienation, and an increase in their feeling of social support from their family are also in line with the overall goals in FFT treatment and may also be important considering the large body of research suggesting that this may be protective factors with respect to development of youth delinquency [16, 17]. The lack of significant changes on the majority of outcomes is, however, important to note. We did not find any changes in parent or youth reports of positive involvement or positive parenting, both parenting skills that are expected to be related to better outcomes later in life [16]. Neither did the youths report changes on the specific dimensions of attachment such as trust and communication in the family. Parents did also not report changes in how disagreement was handled. In summary, these findings for the entire sample indicate that, although some important aspects of family functioning improved for the whole sample, other outcomes expected to be related to adolescent disruptive behavior did not show improvement after treatments.

### Limitations and future directions

This study has several limitations worth mentioning. We experienced a substantial drop-out, and although we employed state-of-the-art techniques to examine and handle missing in our modelling, there may still be ways missingness is affecting our estimates that we are unable to detect. With respect to the external validity of the results, there are also limitations. First, this study covered only the middle, southern, and central part of Norway, and we do not have any way of assessing to what degree the families recruited, or treatment available to the control group, are representative for the Norwegian CWS context. Second, as previously discussed, the sample displayed lower levels of disruptive behavior than anticipated based on the inclusion criteria. This may be related to the population we drew from, but it may also be the result of unintended selection processes into the study (see [31]). Finally, the substantial heterogeneity within the control group, as well as the limited information available on the specific treatments provided, including missing treatment-type information for some cases, constrain our ability to interpret the findings. Although the variability in treatment reflects our intention to evaluate FFT under real-world conditions in Norway, the absence of more comprehensive TAU description limits the extent to which differences between the FFT and control groups can be meaningfully understood.

There are several important future directions that can be drawn from this study. First, with respect to conducting effectiveness studies in regular services, future studies would benefit from gathering more detailed information about the services received by control group participants. Having a clearer picture of what constitutes TAU would strengthen the interpretation of results and provide a more accurate understanding of the contrast between intervention and control conditions in real-world service settings. In addition, monitoring of enrolment should be planned with care when conducting research in regular service settings. Information on enrollment can enable detection of problems with study progress but may also yield valuable insights into sample characteristics and recruitment practices in regular services.

Finally, given that family functioning is consistently found to be related to important life and behavioral outcomes for adolescents, it is important to examine further how treatment can change such factors for families involved with CWS. For FFT, the results suggest that the specific therapeutic elements targeting family functioning should be further examined, especially when FFT is used in settings where the severity of problems may be lower than in juvenile justice involved populations. The results also highlight the importance of including both parents and youths as respondents on family functioning.

## Conclusion

We did not find support for the hypothesized superiority of FFT over treatment as usual (TAU) on family functioning outcomes in a Norwegian context. Findings in the overall sample, regardless of what treatment the families received, however suggest that treatment may improve some important aspects of family functioning, but that improvements may have been limited in scope.

## Supplementary Information

Supplementary Material 1

## Data Availability

Data is not available due to its sensitivity and legal restrictions posed by Norwegian law on sharing personal data without explicit consent from participants. Other materials are available upon request to the corresponding author.

## Footnotes

1 In the current study, the parents were asked the gender of their children, and their options were limited to male or female. As a result, we are limited to what gender parents have ascribed to the youth, and cannot present more comprehensive information about the gender composition of our sample.

2 At the start of the study, a criterion of a score on Youth Level of Services (YLS) of 9 or above was required to indicate risk level. However, one year into the study this criterion was removed as FFT-leaders tasked with assessing inclusion reported that referrals did not contain detailed enough information to score YLS.

3 Drug abuse could not be the sole reason for referral; drug abuse would have to be a secondary problem related to one of the other criteria.

4 Change 1 parameter estimates were calculated as pretest - posttest, while change 2 parameter estimates were calculated as follow-up - posttest. A negative ES for change 1 indicates an increase and a positive ES a decrease, while for change 2 a negative ES indicates a downward change and a positive ES indicate an upward change.

5 One significant difference was found at posttest (intercept) for parent reported conflict in favor of the control group. There was however no significant difference in change between pretest and posttest (change 1). The intercept parameter is a result of the parametrization that allows us to examine change separately in the two different time periods and does not include any control for chance pretest differences. Therefore, we do not treat it as an estimate of effect of treatment.
