# Supplementary material for: Results from a randomized controlled trial of functional family therapy in Norway: effects on family functioning outcomes
Source: Child Adolesc Psychiatry Ment Health. 2026 Jan 17;20:20. doi: 10.1186/s13034-026-01025-4 (PMC12895778; doi:10.1186/s13034-026-01025-4)
Supplement: Supplementary file 1 — Supplementary Material 1 [file 13034_2026_1025_MOESM1_ESM.docx]

**Results from a Randomized Controlled Trial of Functional Family Therapy in Norway: Effects on Family Functioning Outcomes
Supplemental Information**

Asgeir Røyrhus Olseth^12^, Gunnar Bjørnebekk^12^, Serap Keles^3^ and Kristine Amlund Hagen^1^

^1^Norwegian Center for Child Behavioral Development, Oslo, Norway

^2^Department of Special Needs Education, University of Oslo, Oslo, Norway

^3^Knowledge Centre for Education, University of Stavanger, Stavanger, Norway

**Table of contents**

[S11: Calculation of Effect Sizes (Cohen’s *d*) 2](#_Toc214624555)

[SI2: Software Used in the Analyses 3](#_Toc214624556)

[SI3: Psychological Aggression Outcome 4](#_Toc214624557)

[Table S1 4](#_Toc214624558)

[Figure S1 4](#_Toc214624559)

[Figure S2 5](#_Toc214624560)

[Figure S3 5](#_Toc214624561)

[SI4: Missing Data 6](#_Toc214624562)

[Table S2 6](#_Toc214624563)

[Table S3. 6](#_Toc214624564)

[SI5: Auxiliary Variables 8](#_Toc214624565)

[Table S4 8](#_Toc214624566)

[SI6: Model Fit 11](#_Toc214624567)

[Table S5 11](#_Toc214624568)

[SI7 – CONSORT checklist 13](#_Toc214624569)

[References 16](#_Toc214624570)

# S11: Calculation of Effect Sizes (Cohen’s *d*)

Cohen´s *d* was calculated as follows:

1. Unconditional models: *d* = $\frac{Latent mean}{\sqrt{\varphi_{int+ \theta_{1}}}}$
2. Conditional models: *d* = $\frac{B}{\sqrt{\psi_{int}+\theta_{1}}}$

Where *latent mean* is the model estimated means of the change 1 and change 2 parameters from unconditional, and *B* is the estimates for the difference in the intercept and the change parameters. The pooled *SD* at pretest was calculated based on the estimates for the latent intercept variance ${(\varphi}_{int})$ in unconditional models and the residual variance ($\psi_{int}$) in conditional models and the residual variance at T1 ($\theta_{1})$.

For the model where covariance between pretest and follow-up time-specific residuals were freely estimated, the denominator SD in Cohen´s *d* was calculated with the following elements added to the latent intercept variance/residual variance: $\theta_{1}+\theta_{3}-2 \sigma_{13}$ in accordance with the calculation of SD of the difference score when there is a correlation between the two sets of measurements (Lakens, 2013).

# SI2: Software Used in the Analyses

Analyses were conducted in R version 4.2.3 and Mplus version 8.3 (Muthén & Muthén, 2017). Missing data analyses and selection of the auxiliary variables were conducted using the R-packages Lavaan 0.6.15 (Rosseel, 2012) and SemTools version 0.5.6 (Jorgensen et al., 2022). The R-package MplusAutomation version 1.1.1 (Hallquist & Wiley, 2018) was used for automation of modeling in Mplus. G*POWER version 3.1 (Faul et al., 2007) was used for the power analyses.

# SI3: Psychological Aggression Outcome

Table S1 contains estimates of skewness and kurtosis, and figures S1-S3 show distribution of the outcome variable across timepoints. The degree of non-normality and lack of variation in scores above 0, in combination with high degree of missingness over time, led us to abandon the analyses of this outcome.

## Table S1

*Skewness and Kurtosis of the Psychological Aggression Outcomes*

| Variable and timepoint | *n* | Skewness | Kurtosis |
| --- | --- | --- | --- |
| Psychological aggression at pretest | 95 | 3.17 | 11.05 |
| Psychological aggression at posttest | 65 | 6.18 | 41.25 |
| Psychological aggression at follow-up | 58 | 3.08 | 8.69 |

## Figure S1

*Scores on the Psychological Aggression Outcome - Pretest
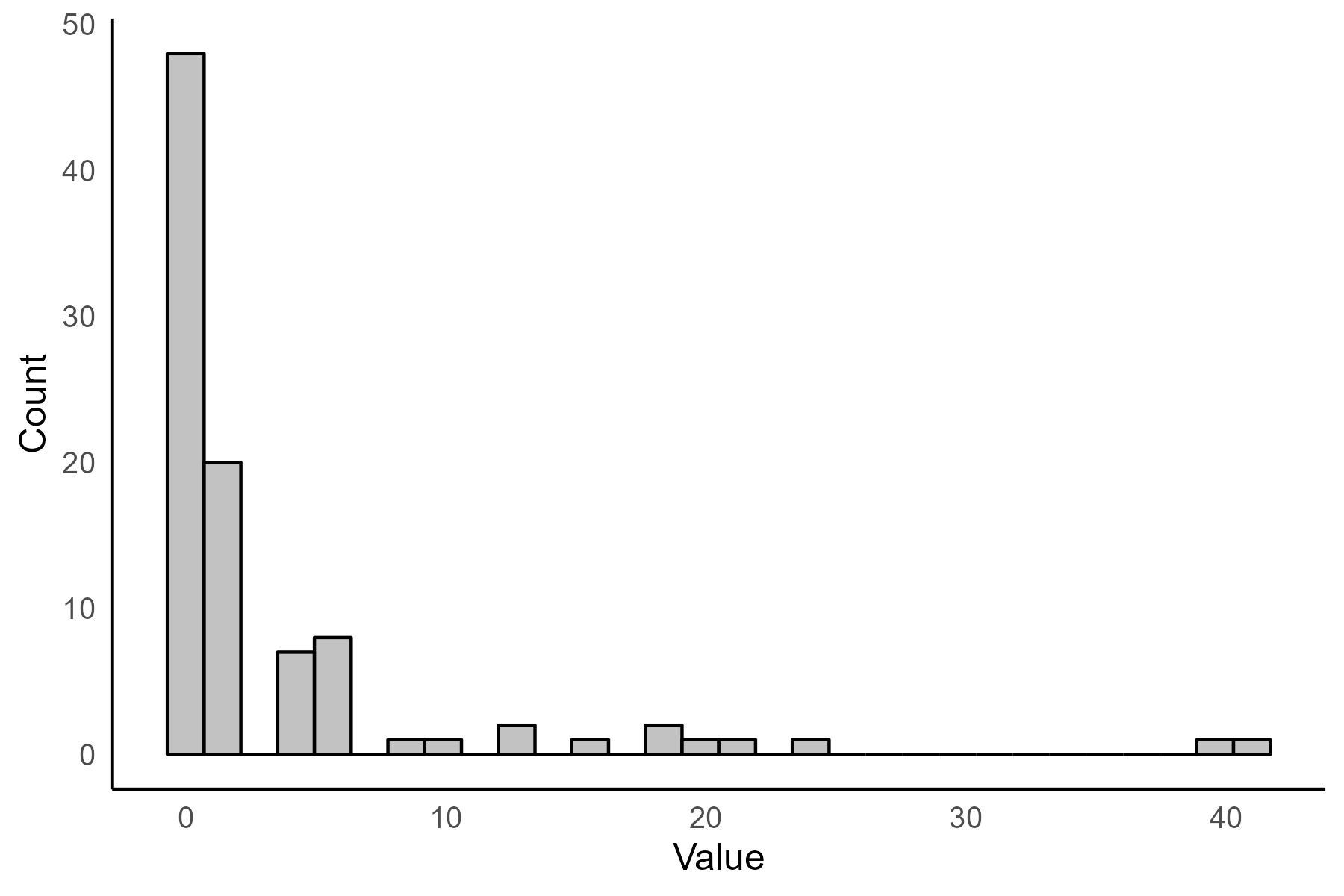
*

## Figure S2

*Scores on the Psychological Aggression Outcome - Posttest
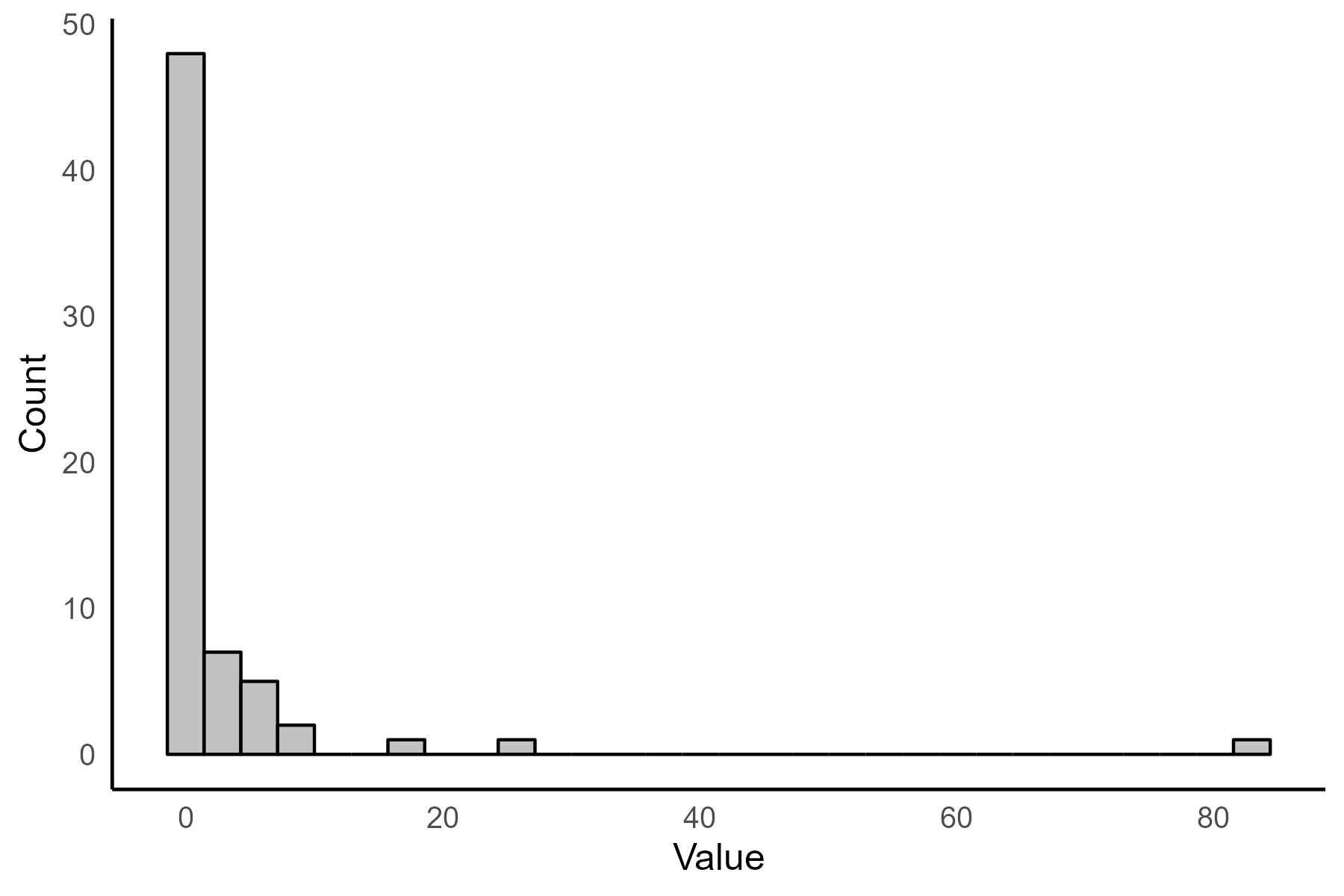
*

## Figure S3

*Scores on the Psychological Aggression Outcome – Follow-up*

*
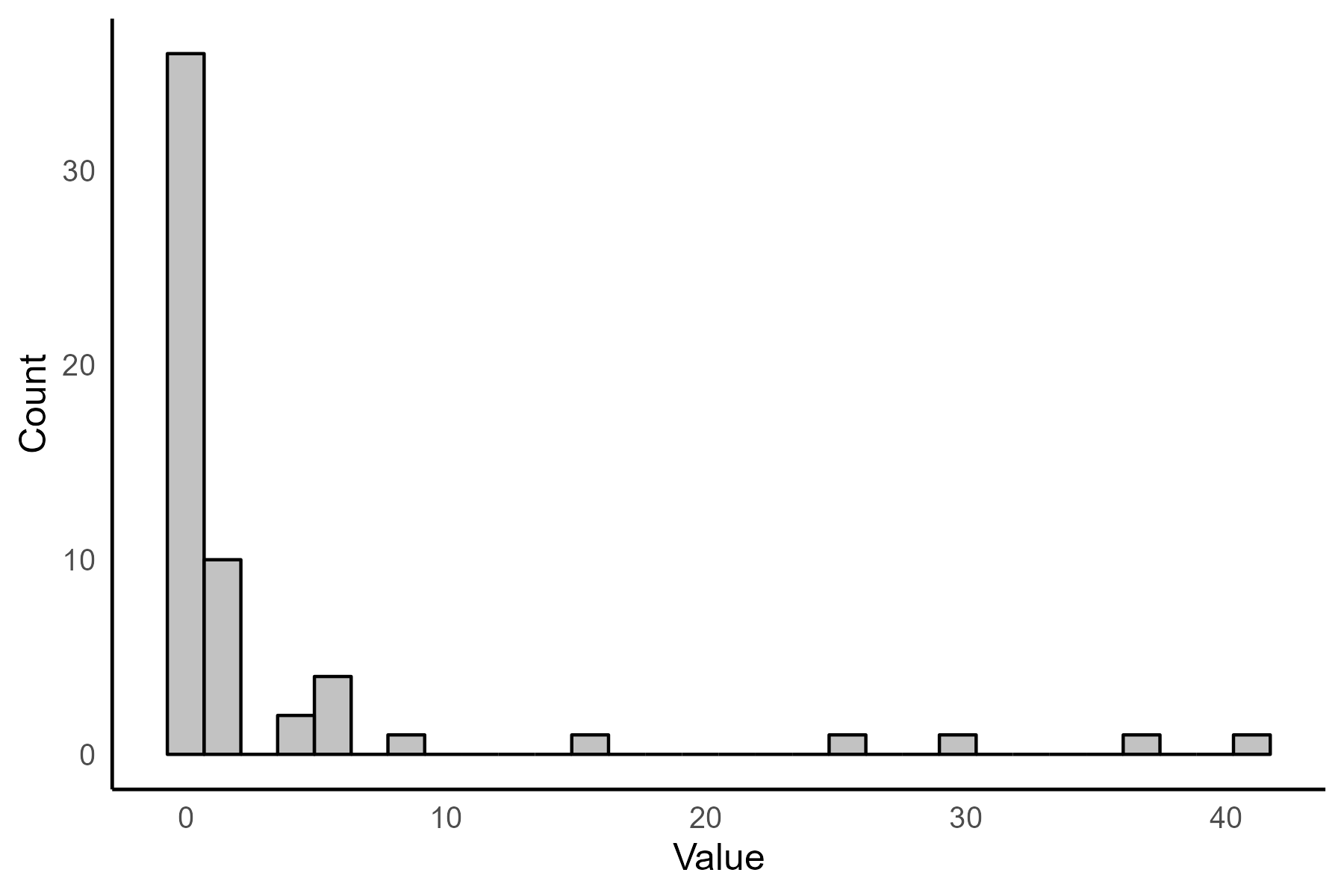
*

# SI4: Missing Data

## Table S2

*Descriptive Statistics - Patterns of Missing Data*

|  | Missing Pattern  Pretest – Posttest – Follow-up | Frequency (%) |
| --- | --- | --- |
| Parent-reported outcomes | | |
|  | 1-1-1 | 97 (61,0%) |
|  | 1-0-0 | 41 (25,8%) |
|  | 1-1-0 | 11 (6,9%) |
|  | 1-0-1 | 9 (5,7%) |
|  | 0-1-1 | 1 (0,6%) |
|  | Total | 159 |
| Youth-reported outcomes | | |
|  | 1-1-1 | 84 (52,8%) |
|  | 1-0-0 | 43 (27,0%) |
|  | 1-1-0 | 16 (10,1%) |
|  | 1-0-1 | 13 (8,2%) |
|  | 0-0-0 | 2 (1,3%) |
|  | 0-0-1 | 1 (0,6%) |
|  | Total | 159 |

*Note.* Missing=0, Present=1.

## Table S3.

*Missing Data Classified by Type of Pattern*

|  | Missing category | Frequency (%) |
| --- | --- | --- |
| Parent-reported outcomes | | |
|  | Present or ignorable missingness | 107 (67.3%) |
|  | Monotone missing after pre-test | 41 (25.8%) |
|  | Missing only at follow-up | 11 (6.9%) |
|  | Total | 159 |
| Youth-reported outcomes | | |
|  | Present or ignorable missingness | 98 (61.6%) |
|  | Monotone missing after pretest | 43 (27,0%) |
|  | Missing only at follow-up | 16 (10,1%) |
|  | Missing All | 2 (1,3%) |
|  | Total | 159 |

*Note.* Classification is based on patterns that may be particularly detrimental to estimates of effect due to selective attrition. This includes monotone patterns of missingness such as the “1-0-0” or “1-1-0” that indicates drop out before one of the end points.

# SI5: Auxiliary Variables

Table S4 lists variables that were included as auxiliary variables in the multiple imputation. Gender and APQ positive parenting at pretest were included in all models as gender was related to missingness in general, and APQ positive parenting at pretest was found to potentially be related to differential drop out. Other potential auxiliary variables were identified by examining standardized mean differences (SMD) on available pretest variables and semi-partial correlations between candidate auxiliary variables and outcomes in each model separately (Raykov & Marcoulides, 2013; Raykov & West, 2015). Criterion used for identification of auxiliary variables were: 1) SMD greater than 0.2 or less than -0.2, and 2) a semi-partial correlation greater than .3 or less than -.3 as suggested by Enders (2022). All family functioning outcomes, with the exception of the Conflict Tactics Scale (CTS; i.e., negotiation and psychological aggression) were examined as potential auxiliary variables. CTS variables were not included due to a particularly high degree of missingness and severe non-normality. In addition, we examined youth age, and two variables found to be related to potential differential drop out in a previous article (pretest rule-breaking behavior and social skills; see Olseth et al., 2024).

## Table S4

*Variables Included as Auxiliary in Multiple Imputation Step*

| **Outcome model and respondent** | **Auxiliary variables** |
| --- | --- |
| Parent-reported outcomes |  |
| Positive involvement | Gender, APQ Positive parenting (Y) T1  APQ Positive parenting (P) T1  APQ Positive parenting (P) T2  APQ Positive parenting (P) T3 |
| Positive parenting | Gender, APQ Positive parenting (Y) T1  APQ Positive involvement (P) T1  APQ Positive involvement (P) T3 |
| Negotiation | Gender  APQ Positive parenting (Y) T1  FES Conflict (P) T2  FES Cohesion (P) T2 |
| Psychological aggression | Gender  APQ Positive parenting (Y) T1  FES Conflict (Y) T1  Social support (Y) T1 |
| Cohesion | Gender  APQ Positive parenting (Y) T1  APQ Positive involvement (P) T1 |
| Conflict | Gender,  APQ Positive parenting (Y) T1  FES Cohesion (P) T1 |
| Youth-reported outcomes |  |
| Positive involvement | Gender  APQ Positive parenting (Y) T1  IPPA Trust (Y) T2  IPPA Alienation (Y) T3  APQ Positive parenting (Y) T3  FES Cohesion (P) T3 |
| Positive parenting | Gender, IPPA  Trust (Y) T2  FES Conflict (Y) T3 |
| Social support | Gender  APQ Positive parenting (Y) T1  IPPA Alienation (Y) T1  APQ Positive involvement (Y) T3  IPPA Trust (Y) T2  FES Cohesion (Y) T2  FES Conflict (Y) T3 |
| Cohesion | Gender  APQ Positive parenting (Y) T1  IPPA Alienation (Y) T1  IPPA Trust (Y) T2  APQ Positive parenting (Y) T3 |
| Conflict | Gender  APQ Positive parenting (Y) T1  IPPA Alienation (Y) T1  IPPA Trust (Y) T2  FES Cohesion (Y) T2  IPPA Alienation (Y) T3  APQ Positive parenting (Y) T3  APQ Positive involvement (Y) T3 |
| Alienation | Gender  APQ Positive parenting (Y) T1  IPPA Trust (Y) T2  FES Cohesion (Y) T2  FES Conflict (Y) T3  APQ Positive parenting (Y) T3 |
| Communication | Gender  APQ Positive parenting (Y) T1  IPPA Alienation (Y) T1  IPPA Alienation (Y) T2  IPPA Trust (Y) T2  IPPA Alienation (Y) T3  APQ Positive parenting (Y) T3 |
| Trust | Gender  APQ Positive parenting (Y) T1  IPPA Alienation (Y) T1  FES Cohesion (Y) T2  IPPA Alienation (Y) T3  FES Conflict (Y) T3  APQ Positive parenting (Y) T3 |

*Note.* APQ = Alabama Parenting Questionnaire. FES = Family Environment Scale. IPPA = Inventory of Parent and Peer Attachment. T1 = pretest, T2 = posttest, T3 = follow-up. Y = Youth. P = Parent.

# SI6: Model Fit

Table S5 contains fit indices for both conditional and unconditional models. Fit was primarily assessed based on Chi-squared test (χ2) of model fit. Root mean square error of approximation (RMSEA) and Comparative fit index (CFI) were reported, but as we examined the models with few degrees of freedom we did not rely on these indices in evaluation of model fit (see Kenny et al., 2014; and Shi et al., 2021, for details on performance of other fit indices under conditions such as these). For the models where χ2 indicated poor model fit, we examined correlations between latent variables as suggested by Mun et al. (2009). See also Olseth et al. (2024) for additional information.

## Table S5

*Model Fit Indices for Conditional and Unconditional LCM*

| Outcome - model | χ2 (*df*) | RMSEA  [90% CI] | CFI |
| --- | --- | --- | --- |
| Parent-reported outcomes | | | |
| Positive involvement- unconditional model^a^ | 0.187 (1) | 0 [0, .159] | 1 |
| Positive involvement - conditional model^a^ | 0.192 (1) | 0 [0, .160] | 1 |
| Positive parenting - unconditional model | 3.690 (2) | 0.073 [0, .189] | .977 |
| Positive parenting - conditional model | 3.759 (2) | 0.074 [0, .190] | .977 |
| Negotiation - unconditional model | 0.066 (2) | 0 [0, 0] | 1 |
| Negotiation - conditional model | 0.078 (2) | 0 [0, 0] | 1 |
| Psychological aggression - unconditional model^b^ |  |  |  |
| Psychological aggression - conditional model^b^ |  |  |  |
| Cohesion - unconditional model | 5.157 (2) | 0.100 [0, 0.210] | .942 |
| Cohesion - conditional model | 5.308 (2) | 0.102 [0, 0.212] | .947 |
| Conflict - unconditional model | 3.781 (2) | 0.075 [0, 0.190] | .979 |
| Conflict - conditional model | 3.471 (2) | 0.068 [0, 0.185] | .986 |
| Youth-reported outcomes | | | |
| Positive involvement- unconditional model | 2.692 (2) | 0.047 [0, 0.171] | . 981 |
| Positive involvement - conditional model | 2.740 (2) | 0.048 [0, 0172] | .981 |
| Positive parenting - unconditional model | 1.959 (2) | 0 [0, 0.156] | 1 |
| Positive parenting - conditional model | 1.649 (2) | 0 [0, 0.148] | 1 |
| Social support - unconditional model | 5.779 (2) | 0.109 [0, 0.218] | .850 |
| Social support - conditional model | 5.891 (2) | 0.111 [0, 0.219] | .856 |
| Cohesion - unconditional model | 0.420 (2) | 0 [0, 0.096] | 1 |
| Cohesion - conditional model | 0.412 (2) | 0 [0, 0.096] | 1 |
| Conflict - unconditional model | 0.481 (2) | 0 [0, 0.101] | 1 |
| Conflict - conditional model | 0.419 (2) | 0 [0, 0.096] | 1 |
| Alienation - unconditional model | 0.247 (2) | 0 [0, 0.075] | 1 |
| Alienation - conditional model | 0.266 (2) | 0 [0, 0.078] | 1 |
| Communication - unconditional model | 0.541 (2) | 0 [0, 0.106] | 1 |
| Communication - conditional model | 0.568 (2) | 0 [0, 0.108] | 1 |
| Trust - unconditional model | 0.014 (2) | 0 [0, 0] | 1 |
| Trust - conditional model | 0.038 (2) | 0 [0, 0] | 1 |

*Note.* RMSEA = root mean square error of approximation; CFI = comparative fit index.

^a^ For this model correlations between time specific residual variances at pretest and follow-up were freely estimated using one additional *df*.

^b^ Psychological aggression was not possible to analyze using LCM and MI, see SI3 for details.

# SI7 – CONSORT checklist

| Section/Topic | Item No | Checklist item | Reported on page No |
| --- | --- | --- | --- |
| Title and abstract | | | |
|  | 1a | Identification as a randomised trial in the title | 1 |
|  | 1b | Structured summary of trial design, methods, results, and conclusions (for specific guidance see CONSORT for abstracts) | 3 |
| Introduction | | | |
| Background and objectives | 2a | Scientific background and explanation of rationale | 5-7 |
|  | 2b | Specific objectives or hypotheses | 8-9 |
| Methods | | | |
| Trial design | 3a | Description of trial design (such as parallel, factorial) including allocation ratio | 12 |
|  | 3b | Important changes to methods after trial commencement (such as eligibility criteria), with reasons | 11-12, previous publication |
| Participants | 4a | Eligibility criteria for participants | 10-11 |
|  | 4b | Settings and locations where the data were collected | 13 |
| Interventions | 5 | The interventions for each group with sufficient details to allow replication, including how and when they were actually administered | 13-14 |
| Outcomes | 6a | Completely defined pre-specified primary and secondary outcome measures, including how and when they were assessed | 9, 13, 15-17 |
|  | 6b | Any changes to trial outcomes after the trial commenced, with reasons | 11-12, previous publication |
| Sample size | 7a | How sample size was determined | 11 |
|  | 7b | When applicable, explanation of any interim analyses and stopping guidelines | N/A |
| Randomisation: |  |  |  |
| Sequence generation | 8a | Method used to generate the random allocation sequence | 12 |
|  | 8b | Type of randomisation; details of any restriction (such as blocking and block size) | 12 |
| Allocation concealment mechanism | 9 | Mechanism used to implement the random allocation sequence (such as sequentially numbered containers), describing any steps taken to conceal the sequence until interventions were assigned | 12 |
| Implementation | 10 | Who generated the random allocation sequence, who enrolled participants, and who assigned participants to interventions | 12 |
| Blinding | 11a | If done, who was blinded after assignment to interventions (for example, participants, care providers, those assessing outcomes) and how | 12 |
|  | 11b | If relevant, description of the similarity of interventions | N/A |
| Statistical methods | 12a | Statistical methods used to compare groups for primary and secondary outcomes | 17, supplemental materials |
|  | 12b | Methods for additional analyses, such as subgroup analyses and adjusted analyses | N/A |
| Results | | | |
| Participant flow (a diagram is strongly recommended) | 13a | For each group, the numbers of participants who were randomly assigned, received intended treatment, and were analysed for the primary outcome | Figure 1 |
|  | 13b | For each group, losses and exclusions after randomisation, together with reasons | Figure 1 |
| Recruitment | 14a | Dates defining the periods of recruitment and follow-up | 10, 12-13 |
|  | 14b | Why the trial ended or was stopped | 11 |
| Baseline data | 15 | A table showing baseline demographic and clinical characteristics for each group | Table 1 |
| Numbers analysed | 16 | For each group, number of participants (denominator) included in each analysis and whether the analysis was by original assigned groups | 17, Figure 1, table 2 and table 3. |
| Outcomes and estimation | 17a | For each primary and secondary outcome, results for each group, and the estimated effect size and its precision (such as 95% confidence interval) | Table 3 |
|  | 17b | For binary outcomes, presentation of both absolute and relative effect sizes is recommended | N/A |
| Ancillary analyses | 18 | Results of any other analyses performed, including subgroup analyses and adjusted analyses, distinguishing pre-specified from exploratory | N/A |
| Harms | 19 | All important harms or unintended effects in each group (for specific guidance see CONSORT for harms) | N/A |
| Discussion | | | |
| Limitations | 20 | Trial limitations, addressing sources of potential bias, imprecision, and, if relevant, multiplicity of analyses | 23-24 |
| Generalisability | 21 | Generalisability (external validity, applicability) of the trial findings | 23-24 |
| Interpretation | 22 | Interpretation consistent with results, balancing benefits and harms, and considering other relevant evidence | 23, 25 |
| Other information | | |  |
| Registration | 23 | Registration number and name of trial registry | 4, references |
| Protocol | 24 | Where the full trial protocol can be accessed, if available | N/A |
| Funding | 25 | Sources of funding and other support (such as supply of drugs), role of funders | Author notes |

*Note:* SM = Supplemental material. N/A = Not applicable. Consolidated Standards of Reporting Trials checklist (Schulz et al., 2010).

# References

Enders, C. K. (2022). *Applied missing data analysis* (2nd ed.). Guilford Press.

Faul, F., Erdfelder, E., Lang, A. G., & Buchner, A. (2007). G*Power 3: a flexible statistical power analysis program for the social, behavioral, and biomedical sciences. *Behavior Research Methods*, *39*(2), 175-191. https://doi.org/10.3758/bf03193146

Hallquist, M. N., & Wiley, J. F. (2018). MplusAutomation: An R package for facilitating large-scale latent variable analyses in mplus. *Structural Equation Modeling: A Multidisciplinary Journal*, *25*(4), 621-638. https://doi.org/10.1080/10705511.2017.1402334

Jorgensen, T. D., Sunthud, P., Schoemann, A. M., & Rosseel, Y. (2022). s*emTools: Useful tools for structural equation modeling (Version 0.5.6) [R package].* In https://CRAN.R-project.org/package=semTools

Kenny, D. A., Kaniskan, B., & McCoach, D. B. (2014). The performance of RMSEA in models with small degrees of freedom. *Sociological Methods & Research*, *44*(3), 486-507. https://doi.org/10.1177/0049124114543236

Lakens, D. (2013). Calculating and reporting effect sizes to facilitate cumulative science: a practical primer for t-tests and ANOVAs. *Frontiers in Psychology*, *4*, 863. https://doi.org/10.3389/fpsyg.2013.00863

Mun, E. Y., von Eye, A., & White, H. R. (2009). An SEM Approach for the Evaluation of Intervention Effects Using Pre-Post-Post Designs. *Structural Equation Modeling: A Multidisciplinary Journal*, *16*(2), 315-337. https://doi.org/10.1080/10705510902751358

Muthén, L. K., & Muthén, B. O. (2017). *Mplus User’s Guide* (8th ed.), Muthén & Muthén.

Olseth, A. R., Hagen, K. A., Keles, S., & Bjornebekk, G. (2024). Functional family therapy for adolescent disruptive behavior in Norway: Results from a randomized controlled trial. *Journal of Family Psychology*, *38*(4), 548-558. https://doi.org/10.1037/fam0001213

Raykov, T., & Marcoulides, G. A. (2013). Identifying useful auxiliary variables for incomplete data analyses. *Educational and Psychological Measurement*, *74*(3), 537-550. https://doi.org/10.1177/0013164413511326

Raykov, T., & West, B. T. (2015). On enhancing plausibility of the missing at random assumption in incomplete data analyses via evaluation of response-auxiliary variable correlations. *Structural Equation Modeling: A Multidisciplinary Journal*, *23*(1), 45-53. https://doi.org/10.1080/10705511.2014.937848

Rosseel, Y. (2012). lavaan: An R package for structural equation modeling. *Journal of Statistical Software*, *48*(2), 1 - 36. https://doi.org/10.18637/jss.v048.i02

Schulz, K. F., Altman, D. G., Moher, D., & Group, C. (2010). CONSORT 2010 statement: updated guidelines for reporting parallel group randomised trials. *BMC Med*, *8*, Article 18. https://doi.org/10.1186/1741-7015-8-18

Shi, D., DiStefano, C., Maydeu-Olivares, A., & Lee, T. (2021). Evaluating SEM model fit with small degrees of freedom. *Multivariate Behavioral Research*, 1-36. https://doi.org/10.1080/00273171.2020.1868965
